# Intracardiac versus transesophageal echocardiography for diagnosis of left atrial appendage thrombosis in atrial fibrillation: A meta‐analysis

**DOI:** 10.1002/clc.23698

**Published:** 2021-09-03

**Authors:** Guijun He, Hanxion Liu, Xiaoyu Huang, Xiaoqi Deng, Guosu Yang, Duan Luo, Lin Cai

**Affiliations:** ^1^ Chengdu Third People's Hospital, Chengdu Institute of Cardiovascular Diseases Chengdu China; ^2^ Fourth People's Hospital of Chengdu Chengdu China

**Keywords:** atrial fibrillation, intracardiac echocardiography, thrombosis, transesophageal echocardiography

## Abstract

**Introduction:**

Left atrial appendage (LAA) thrombus in patients with atrial fibrillation is usually detected by transesophageal echocardiography (TEE). Intracardiac echocardiography (ICE) can be a suitable alternative to detect thrombosis. However, the effectiveness of the two methods for detecting LAA thrombus is still unclear, we performed a meta‐analysis that compared ICE versus TEE for LAA thrombosis.

**Methods:**

We searched PubMed, Cochrane Library, and Embase for published abstracts and manuscripts on June 1, 2020. The analysis was performed using RevMan 5.3, STATA 15, and Meta‐Disc 1.4.

**Results:**

Eight studies consists of 1108 patients (TEE = 558 vs. ICE = 550) were included. The average sensitivity of ICE and TEE to diagnose LAA thrombus is 1.0 (95% CI: 0.91–1.00) versus 0.68 (95% CI: 0.49–0.83), and specificity of ICE and TEE to diagnosis of LAA thrombus is 1.0 (95% CI: 0.99–1.00) versus 0.98 (95% CI: 0.96–0.99). The AUC of ICE and TEE is 0.9846 (SEAUC = 0.0196) and 0.9655 (SEAUC = 0.0401), and the Q* statistics is 0.9462 (SEQ* = 0.0406) and 0.9127 (SEQ * = 0.0616), respectively. Z test was performed on Q* statistics (Z = 0.45, *p* > .05).

**Conclusion:**

The ICE and TEE have similar diagnostic efficacy for LAA thrombosis, but the ICE has higher sensitivity. Compared with TEE, ICE may be more advantages and prospects for clinical application.

## 1 INTRODUCTION

Atrial fibrillation (AF) is the most common arrhythmia, and the proportion increases with age. The proportion of AF is as high as 8% over 75 years of age.^1^ The most effective treatment for AF is radiofrequency ablation and cryoablation, but patients need to exclude the left atrium and left atrial appendage (LAA) thrombosis. The main method is to routinely perform transesophageal echocardiography (TEE) before the operation to exclude LAA thrombus.

Intracardiac echocardiography (ICE) has been increasingly applied to probe the structure of the left atrium and LAA. Currently, the two methods are unclear about the real events of detecting LAA thrombus. Therefore, we performed a meta‐analysis.

## 2 METHODS

### 2.1 Search strategy

After searching PubMed, Cochrane Library, and Embase by using keywords: atrial fibrillation, transesophageal echocardiography, Intracardiac echocardiography, and thrombosis from their inception on June 1, 2020. we found that studies only be published in English language.

### 2.2 Study selection

- The eligibility criteria for our meta‐analysis including: (a) Studies are prospectively or retrospectively including TEE and ICE. (b) The clinical result is the gold standard which results in an uneventful AF ablation. (c) The studies had to provide sufficient information to construct the 2 × 2 contingency table, that is, false and true positives and negatives were provided. If not directly given, get it from the corresponding author via email. (d) TEE and ICE inspection interval is less than 48 h and the definition of thrombosis consistent or similar, and the anticoagulation standard is consistent.
- Exclusion criteria: (a) Data cannot be accurately extracted, and it cannot be obtained from the corresponding author. (b) Animal experiments and review literature. (c) Articles with undetectable thrombus.

### 2.3 Data extractions and quality assessment

Two independent reviewers screened the documents according to the pre‐established inclusion and exclusion criteria and including the documents according to the QUADAS‐2 (quality assessment of diagnostic accuracy studies‐2) evaluation criteria.^2^ Carry out the quality assessment, extract data, and cross‐check. If opinions are inconsistent, the third researcher will make a joint decision. The extracted data includes basic information, experimental design, and original data (true positives, false positives, true negatives, and false negatives).

### 2.4 Statistical analysis

1. Using Q test to detect whether there is heterogeneity, and using I2 to estimate the size of the heterogeneity, and then selecting the appropriate statistical analysis model for subsequent meta‐analysis
2. We tabulated true positives, false negatives, false positives, and true negatives in patients with LAA thrombus in ICE and TEE. The random‐effects model was used to calculate the average sensitivity, specificity, likelihood ratio, and 95% confidence interval (CI) of TEE and ICE respectively. The Mose's constant linear model was used to fit the SROC curve, and the diagnostic odds ratio (DOR), the area under the curve (AUC), and Q * statistics were used to evaluate the accuracy of the diagnostic tests ICE versus TEE in the diagnosis of LAA thrombus.^3^, ^4^ We also used the Z test to analyze whether there are differences between the two diagnostic methods. Calculating the Spearman correlation coefficient *ρ* of true positive rate and false‐positive rate, and analyzing whether there is an threshold effect. Once Q statistic does not prove the heterogeneity is not necessary to look for the Spearman correlation coefficient. Deeks linear regression will be used to assess whether the included studies had publication bias. The statistical software for this article is Review Manager 5.3, STATA 15, and Meta‐Disc 1.4, *p* < .05 is considered statistically significant.

## 3 RESULTS

### 3.1 Search results

**FIGURE 1:**
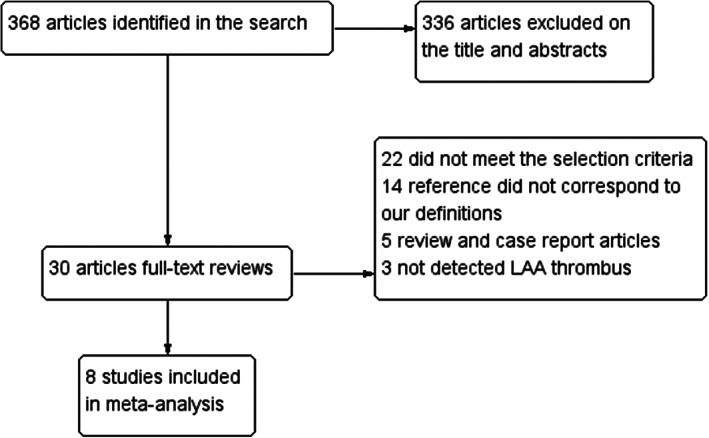
Flow diagram for the included studies

A total of 368 articles were found, 336 articles were excluded from reading titles and abstracts, and 30 articles were initially included (Figure 1). After further reading the full text, we excluded 22 documents that did not meet the inclusion criteria, and finally adopted a total of 8 documents, and recruited 1108 patients (TEE = 558 vs. ICE = 550).^5^, ^6^, ^7^, ^8^, ^9^, ^10^, ^11^, ^12^ QUADAS‐2 quality graph (Figures S2 and S3). Individual study data obtained are given in Table 1. The true positive, false positive, false negative, and true negative of ICE and TEE are shown in (Table S2).

**TABLE 1:** General characteristics of included trials

Author	Year	Type of study	Mean age	Male (%)	Permanent AF (%)	Patients enrolled (ICE vs. TEE)
Saksena et al	2010	Prospective	58	84.2	91	95 vs. 95
Stec et al	2011	Retrospective	49	66.7	25	12 vs. 12
Ren et al	2013	Retrospective	57.8	NA	NA	56 vs. 56
Baran et al	2013	Prospective	54	74	13	76 vs. 76
Anter et al	2014	Prospective	60.5	73	NA	71 vs. 69
Sriram et al	2015	Retrospective	62.6	73.8	29.5	122 vs. 122
Baran et al	2017	Prospective	65	57	57	21 vs. 21
Ikegami et al	2017	Retrospective	69	83	69	97 vs. 107

### 3.2 Heterogeneity test

We have applied DOR as the effect size to analyze the heterogeneity of ICE and TEE, respectively. The Q test proves that Cochran‐Q is 1.75 and 6.15 respectively. That means heterogeneity between studies is small.

**FIGURE 2:**
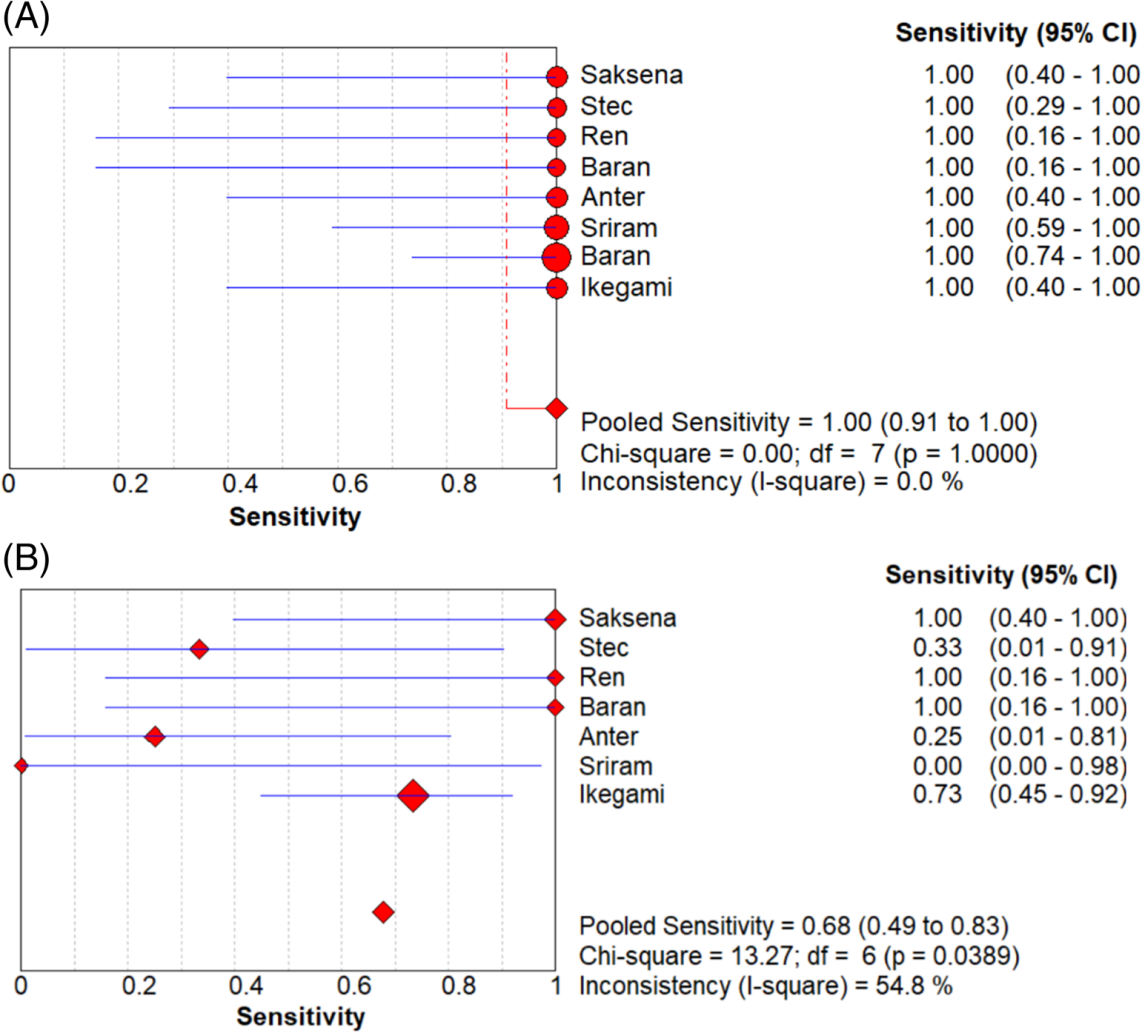
Forest plots of the sensitivity of ICE(A) and TEE (B)

**FIGURE 3:**
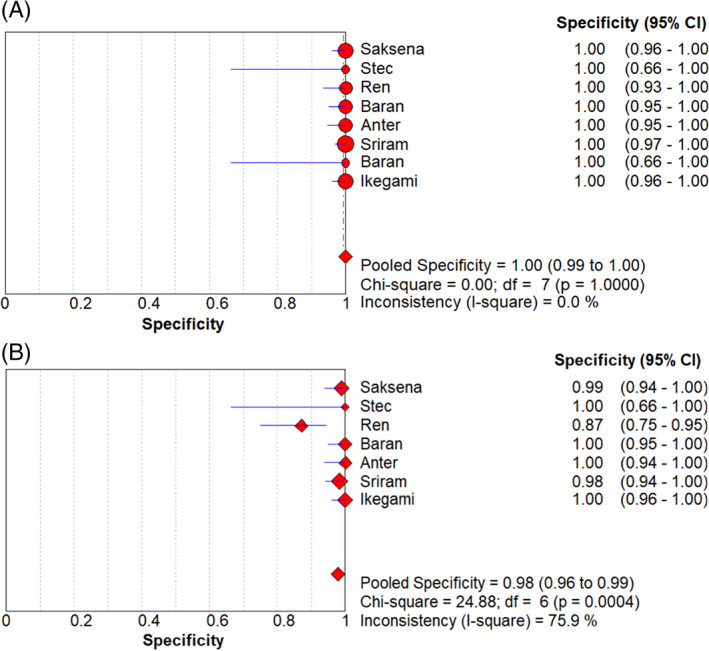
Forest plots of specificity of ICE(A) and TEE (B)

The sensitivity and specificity of the forest plots are shown in Figure 2. The average sensitivity of ICE to diagnose LAA thrombosis was 1.0 (95% CI: 0.91–1.00), and the average sensitivity of TEE was 0.68 (95% CI: 0.49–0.83). Figure 3 shows the ICE and TEE forest plots has been used to detect the specificity of LAA thrombus. The average specificity of ICE in the diagnosis of LAA thrombosis is 1.0 (95% CI: 0.99–1.00), and the average specificity of TEE is 0.98 (95% CI: 0.96–0.99). In addition, the positive likelihood ratio (PLR) of LAA thrombus diagnosed by ICE and TEE is 84.00 (95% CI: 31.56–223.55) versus 25.75 (95% CI: 6.70–98.95); Negative likelihood ratio (NLR) is 0.10 (95% CI: 0.04–0.26) versus 0.47 (95% CI: 0.26–0.86), DOR is 872.70 (95% CI: 208.12–3659.42) versus 89.46 (95% CI: 24.64–324.76), the data is given in Table 2.

**TABLE 2:** Pooled sensitivity, pooled specificity, and pooled likelihood ration of ICE and TEE

	Pooled sensitivity (95%CI)	Pooled specificity (95%CI)	Pooled positive LR (95%CI)	Pooled negative LR (95%CI)	Pooled DOR (95%CI)
ICE	1.0 (0.91–1.00)	1.0 (0.99–1.00)	84.00 (31.56–223.55)	0.10 (0.04–0.26)	872.70 (208.12–3659.42)
TEE	0.68 (0.49–0.83)	0.98 (0.96–0.99)	25.75 (6.70–98.95)	0.47 (0.26–0.86)	89.46 (24.64–324.76)

Abbreviations: CI, confidence interval; DOR, diagnostic odds ratio; LR, likelihood ratio.

The SROC curve of ICE and TEE to diagnose LAA thrombosis is shown in (Figure S6). The AUC of ICE and TEE is 0.9846 (SEAUC = 0.0196) and 0.9655 (SEAUC = 0.0401), and the Q* statistics is 0.9462 (SEQ* = 0.0406) and 0.9127 (SEQ* = 0.0616), respectively. Z test is performed on Q* statistics (Z = 0.45, *p* > .05), and there is no statistical difference between ICE and TEE.

### 3.3 Sensitivity analysis

Sensitivity analysis is performed by reducing one article at a time to assess the impact of a study on the meta‐analysis. There is no difference in results after excluding each article.

### 3.4 Publication bias

We used Deeks to evaluate publication bias for included studies, as shown in (Figure S7). ICE's Deeks linear regression shows that *p* < .05, and we found publication bias. The Deeks linear regression of TEE shows *p* > .05, and no bias was found. ICE publication offset may comes from the search scope which is limited to the published research, and the search for the unpublished research is not performed. (Ethical Approval and Consent to participate: Not applicable. Consent for publication: yes. Code availability: yes).

## 4 DISCUSSION

ICE and TEE mainly evaluating LAA through the following methods: (a) measurement of LAA length, width, and cross‐sectional area; (b) evaluation for thrombus; (c) evaluation for spontaneous echo contrast (SEC), Spontaneous echo contrast (SEC) indicates blood stasis in cardiac chambers and major vessels, and is a known precursor of thrombus formation. TEE plays a pivotal role in detecting and grading SEC in the left atrial (LA) cavity. Assessing LA SEC can identify patients at increased risk for thromboembolic events; (d) measurement of ejection velocities of the appendages by pulsed‐wave Doppler. When be diagnosed as thrombus, TEE and ICE measured similarly low pulsed‐wave Doppler velocities of the LAA (≤20 cm/s). ICE detected a moderate or greater degree of SEC, and had an appendage ejection Doppler velocity ≤20 cm/s as measured by TEE.^9^, ^13^

ICE and TEE have their advantages and limitations. TEE would cause more discomfort for patients, requiring fasting and drinking, and damage to the esophagus. Maltagliati et al. found that TEE has artifacts in LAA, Artifacts were identified in 11 controls (37%); no thrombi were detected during surgical left appendage inspection in these cases. Therefore, TEE could lead to false positive diagnosis of LAA thrombus.^14^ The merit of TEE is low cost. Less pain would be performed at ablation operation, and ICE would guide other intracardiac procedures, such as LAA occlusion, ventricular premature beat positioning, and ventricular septal ablation. The use of ICE catheter ablation of AF is associated with significant fewer major complications and lower fluoroscopy and radiofrequency time.^15^ Friedman et al analyzed predictors of cardiac perforation in a nationwide registry of 102 398 patients undergoing AF ablation. In this registry, ICE had been applied into 73% of patients, and the absence of ICE use was associated with a significantly higher rate of cardiac perforation (odds ratio: 4.85; 95% confidence interval: 4.11–5.71; *p* < .0001).^16^ However, ICE is expensive. In addition, ICE requires further vascular access and young operators need a certain amount of learning time to master this technology. If the ICE is located in the right atrium, there is a distance from the LAA, which may affect its accuracy. Other meta‐analysis studies have shown about the use of ICE and TEE in LAA occlusion, ICE is a feasible and safe alternative that reduces exposure to general anesthesia and associated potential risks.^17^ But Our research focuses on the application of ICE and TEE in thrombosis.

This article conducted a meta‐analysis of the eight included studies, compared with the diagnostic efficacy of ICE and TEE for LAA thrombus by combining diagnostic effect amounts and SROC curves. Finally, the credibility of this meta‐analysis was evaluated by sensitivity analysis and test publication bias. The results be combined with DOR of ICE and TEE were 872.70 and 89.46, respectively, which suggested that both of them have a significant correlation with LAA thrombus, and the correlation of ICE is higher. The SROC curve shows that the AUC of ICE and TEE are 0.9846 (SEAUC = 0.0196) and 0.9655 (SEAUC = 0.0401), and the Q* statistics are 0.9462 (SEQ* = 0.0406) and 0.9127 (SEQ* = 0.0616), respectively. Z test was performed on Q* statistics (Z = 0.45, *p* > .05), and there is no statistical difference between ICE and TEE. It shows that ICE is not significantly better than TEE for LAA thrombus identification ability. The main reasons that why we thought ICE is better than TEE are: (a) TEE probe obviously can be positioned only in the esophagus, whereas the ICE probe can be placed in various sites inside the cardiac chambers, and more conducive to understanding the real situation of LAA thrombus and structure. (b) TEE requires good cooperation from the patients to better observe LAA, and poor synergism may leads to negative results. (c) ICE can reach the left atrium, which is more conducive to find the thrombus hidden between the pectinate muscles. (d) ICE can reduce the number of contrast agents and radiation. The heterogeneity among the studies included in this article is relatively small.

## 5 LIMITATIONS

The number of cases reported in the relevant literature retrieved literature is not large enough, and more randomized controlled trials are needed to verify the reliability of the results. The incidence of LAA thrombotic events is low, which may impacts the interpretability of the meta‐analysis and its ability to detecting differences. Four studies were performed in a retrospective fashion, which might become a limitation of this meta‐analysis. The retrieved literature is not comprehensive. The search scope is limited to the published research, some gray documents in other language studies may be missed. TEE is usually performed prior to ICE. Therefore, in these studies, the ICE operators were blinded to the TEE results. Maybe the 1.0 sensitivity is because the ICE reader already had the TEE results.

## 6 CONCLUSION

In summary, ICE and TEE have similar diagnostic efficacy on LAA thrombosis, but ICE has higher sensitivity. TEE is a choice for those with financial difficulties. ICE may be more appropriate for patients who require transseptal puncture, which has advantages over TEE and has clinical application prospects.

## CONFLICT OF INTEREST

The authors declare no conflicts of interest.

## Supporting information


**Appendix S1**: Supporting information.

## Data Availability

The data that support the findings of this study are available from the corresponding author upon reasonable request.
