# Supplementary material for: Intracardiac versus transesophageal echocardiography for diagnosis of left atrial appendage thrombosis in atrial fibrillation: A meta‐analysis
Source: Clin Cardiol. 2021 Sep 3;44(10):1416–21. doi: 10.1002/clc.23698 (PMC8495084; doi:10.1002/clc.23698)
Supplement: Supplementary file 1 — Appendix S1: Supporting information. [file CLC-44-1416-s001.docx]

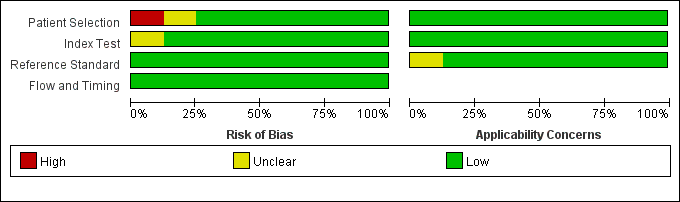


Figure 2:Methodological quality graph.


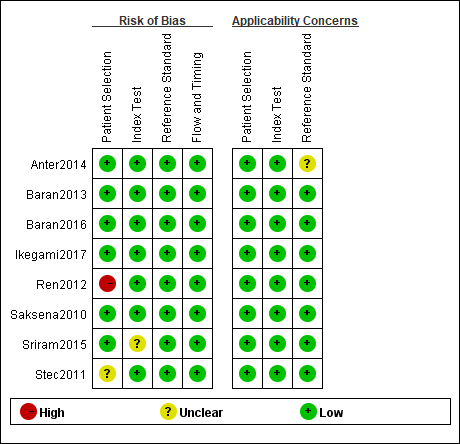


Figure 3:Methodological quality graph


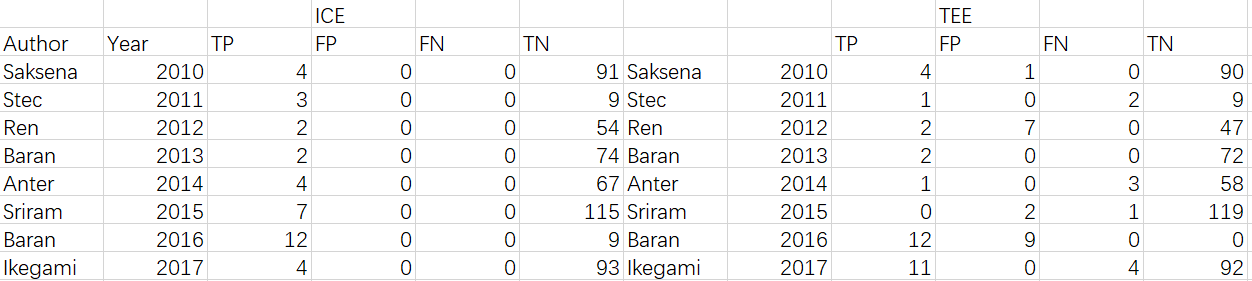


Tab 2 Summary of results of ICE and TEE in included studies, TP=true positive. FP=false positive. TN=true negative. FN=false negative


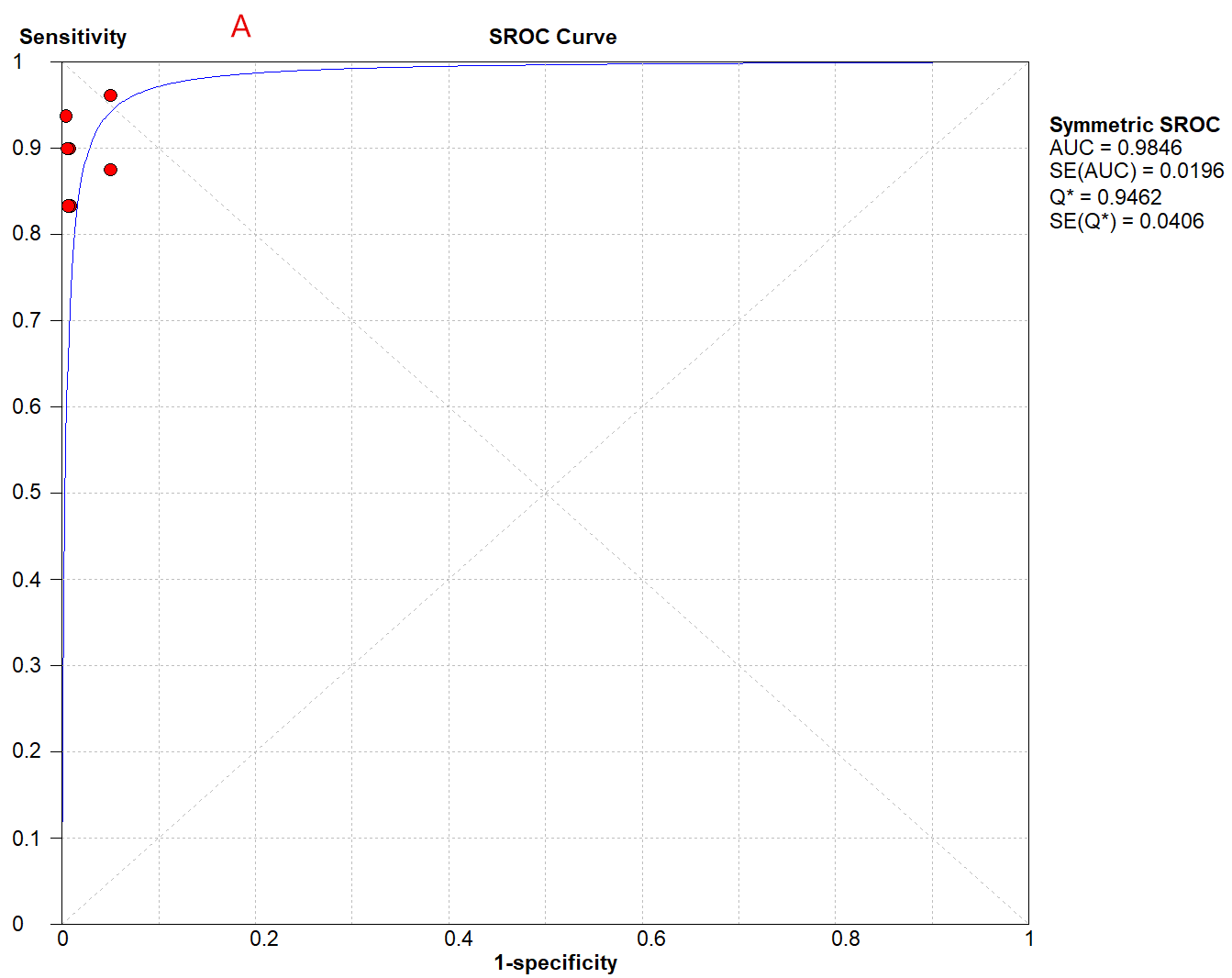

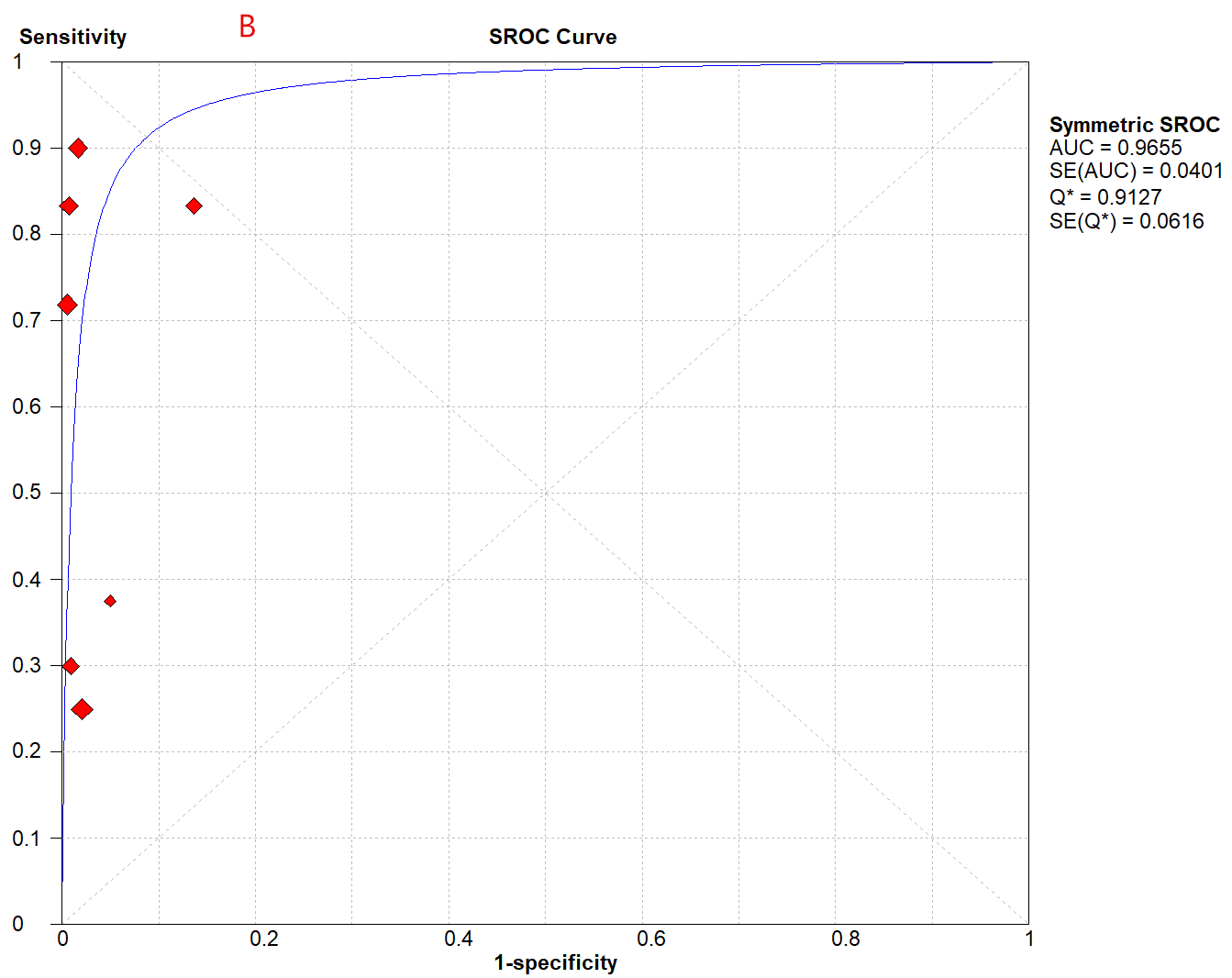


Fig 6 SROC curve of ICE(A) and TEE (B)


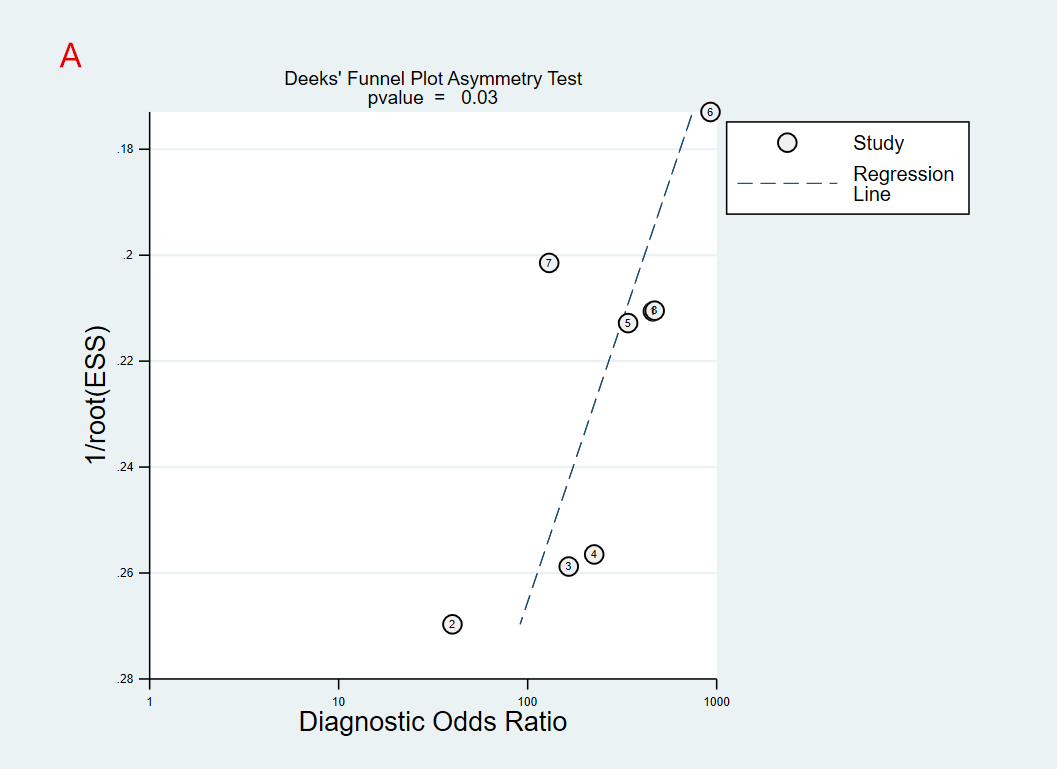

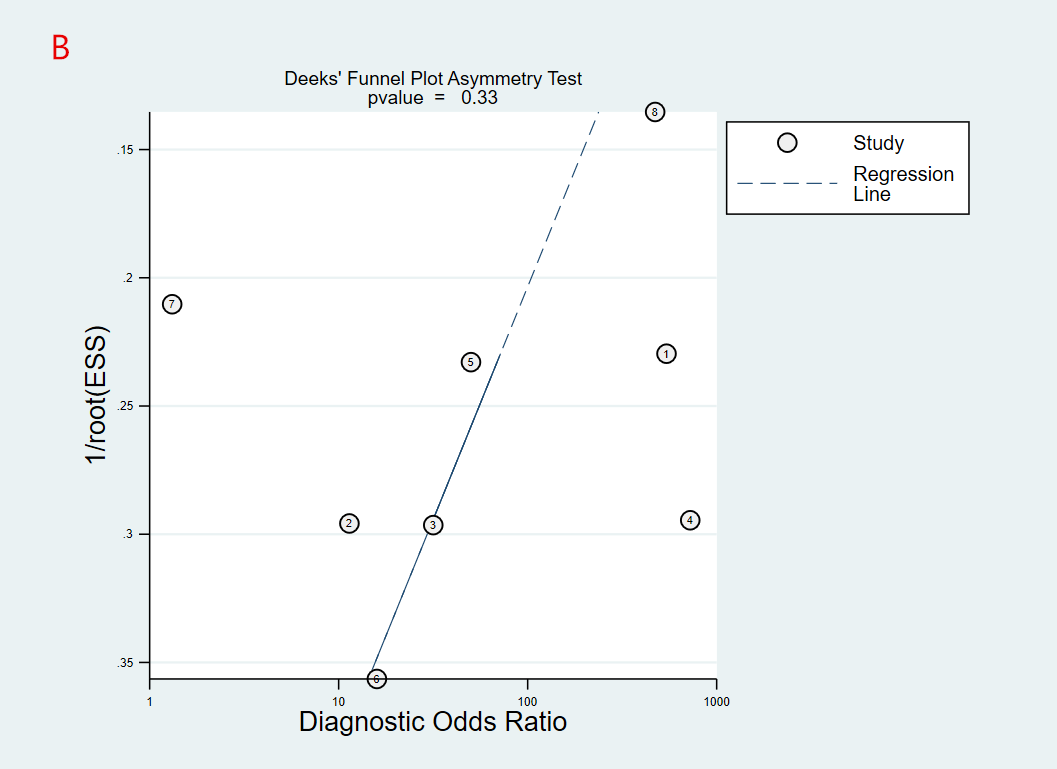
Fig 7 Funnel graph for ICE(A) and TEE (B)
